# Bone Graft

**Published:** 1919

**Authors:** 


**Bone Graft. Abnormal Cases.** By Lt. Julius A. Miller.

In this report the author endeavors to show that under certain conditions where it would have been thought that the graft would be a failure, the results obtained were those expected only in selected cases.

In the instance of one transplant performed on a man who had lost about 3 inches of his right tibia, the scar tissue was so dense and adherent, and covered such a great area of skin, that 3 days after the operation for the repair of the tibia by bone grafting, the entire scar tissue of skin had sloughed away owing to the lack of blood supply. This left about 2 inches of the graft exposed. The upper and lower parts of skin incision, which were beyond the scarred area, united by primary union, and the exposed bone was completely covered by healthy granulations within about ten days. The graft took well and the union was firm. X-ray plate showed at end of two months that union had taken place.

Another case, where in spite of the exposure of the transplanted bone the graft remained and united, was in a man in whom an 8 inch piece of bone taken from the tibia was transplanted into the spinous processes of the man's lumbar vertebrae.

Another interesting transplant of bone in a non-selected case was in an un-united tibia following a compound fracture, where 7 months after the last signs of infection had ceased there were 3 sinuses from fragments of bone embedded in the fractured ends of the tibia. A 6 inch piece of bone was transplanted from the same tibia, and 3 pieces of dead bone from which the sinuses emanated were removed. The area about one of the pieces was well curetted, and the other two were removed by chisel and saw in order to make the bed for the graft. The union took place as if in a selected case.

Conclusions :

1. Exposure of a portion of the transplant bone is not entirely detrimental to the results. The graft will live if the ends of the graft form good union.

2. The presence of a sinus and detritus of bone will not prevent union (sepsis being eliminated) of the graft to its bed.

There are numerous cases in which sinuses are present from embedded pieces of bone or foreign bodies. It would be an irreparable loss of time to the patient if the surgeon should wait in all cases until the discharge from the sinuses had cleared up and remained healed. If there is no further evidence of infection, regardless of the sinuses, an attempt should be made to repair the bone.

